# Sediment loss in response to scheduled pasture ploughing and reseeding: The importance of soil moisture content in controlling risk

**DOI:** 10.1016/j.still.2020.104746

**Published:** 2020-10

**Authors:** S. Pulley, A.L. Collins

**Affiliations:** Sustainable Agriculture Sciences, Rothamsted Research, North Wyke, Okehampton, Devon, EX20 2SB, UK

**Keywords:** Soil erosion, Pasture, Ploughing, Soil moisture, Ruminant farming

## Abstract

Soil water regimes have been shown to have important implications for the erosion risks associated with land management decisions. Despite this, there remains a paucity of information on soil moisture thresholds for farm management operations including the periodic ploughing and reseeding of improved pasture used for ruminant farming. Against this background, this study analysed sediment loss monitored on a heavily instrumented farm platform, in SW England, over four phases of ploughing and reseeding. Precipitation and sediment yields were highly variable between the ten different field scale catchments on the experimental platform after reseeds. Post-plough period rainfall ranged between 461–1121 mm and corresponding sediment yields between 0.20 - 3.13 t. ha^−1^ yr^−1^. The post-plough and reseeding periods accounted for a very high proportion (mean 28.8 %) of monitored sediment fluxes over the study (2012–2019) despite only covering an average of 10.9 % of the 2002 days of flume monitoring. Post-plough sediment yields were highest (2.57 t. ha^−1^ yr^−1^ and 3.13 t. ha^−1^ yr^−1^) when two catchments were ploughed in autumn months and soils were saturated. The yields for the same catchments after summer ploughing were far lower (0.72 t. ha^-1^yr^-1^and 0.73 t. ha^-1^yr^-1^). Thresholds of 35–38 % soil moisture were identified at which ploughing represented a highly elevated erosion risk. Whilst pinpointing thresholds for the clay loam soils with slowly permeable drainage in the study area, the results serve to illustrate the wider need for robust scientific data on soil moisture status to help guide the timing of farm management operations for improving production, to help reduce negative environmental consequences.

## Highlights

• Post-plough sediment yields ranged between 0.20 to 4.73 t. ha yr-1.
• Post-plough periods accounted for a mean of 28.8 % of monitored sediment flux.
• The post-plough periods only covered 10.9 % of the 2002 days of monitoring.
• Critical soil moisture thresholds of between 35–38 % were identified.
• Ploughing soils at or above this threshold resulted in the highest sediment yields.

## 1 Introduction

Increasing demand for food, energy and water in the context of population growth mean that the sustainable use of finite agricultural land resources is now a policy priority (Godfray et al., 2010; McBratney et al., 2014). Here, soil erosion constitutes an important and widespread threat to the sustainability of farmed soils (Boardman and Poesen, 2006; FAO, 2011; Borrelli et al., 2017). Throughout much of the 20th century, erosion studies were primarily focused on the on-site effects of erosion such as physical soil loss, reduction in organic matter and nutrient content and reduced soil fertility (McGonigle et al., 2014), with limited consideration of the off-site impacts and their costs to society. However, in recent years, a process continuum spanning from farm management, to erosion and sediment transport processes, to negative impacts on water quality, to resulting costs to society has also been increasingly recognised and applied in environmental research and policy (Collins and Zhang, 2016).

Grassland utilisation has intensified in many countries, including the UK, as the global demand for animal-derived food products increases (EFMA, 2000; EPA, 2010; Cui et al., 2014). Nationally in the UK, grassland represents 67 % of total agricultural land area meaning that its sustainable management is key to ensuring the maintenance of soil fertility and the good status of waterbodies (Defra, 2015). Soils in the UK under productive grassland with an intact sward experience lower erosion rates when compared to cultivated land, with typical rates ranging between 0.17 and 1.38 t ha^-1^ yr^−1^ (Whitmore et al., 2004; Bilotta et al., 2010; Evans et al., 2017). For comparison, arable soils typically experience higher erosion rates at ∼10 t ha^−1^ yr^−1^ but these rates can be as high as 66 t ha^−1^ yr^−1^ (van Oost et al., 2009). Pasture ploughing and reseeding is a component of the normal management cycle of intensively managed grasslands and one which increases the risk of soil erosion (Evans, 1990). It is typically scheduled to help improve sward quality and productivity (Peel et al., 1985; Hopkins et al., 1990, 1994; Butler and Haygarth, 2007; Drewer et al., 2017; Blair et al., 2018). The average proportion of farm area reseeded annually for grass in the UK has recently been reported as 11.8 %, with a range between 4.2 %–50 % (Agriculture and Horticulture Development Board, 2018).

Whilst ploughing and reseeding can have distinct benefits, sward cover is essential for protecting soil aggregates from detachment and mobilisation (Thompson et al., 2001; Udawatta et al., 2004; Butler and Haygarth, 2007). As a result, ploughed fields would be expected to show erosion characteristics more comparable to arable land until new vegetation cover has established. The presence of vegetation on grassland fields forms a barrier to raindrop impact, breaks up overland flow and reduces slope length (Zuazo and Pleguezuelo, 2008). Vegetation also acts to increase soil aggregate stability and water infiltration rate, reducing erosive overland flows (Morgan and Rickson, 1995).

A preliminary study using field scale monitoring data covering the period 2012–2014 from a heavily instrumented farm platform with temperate grassland fields in South West UK identified that the detachment of soil particles by raindrop-impacted saturation-excess overland flow was the dominant soil erosion mechanism (Pulley and Collins, 2019). As a result, sediment flux scaled linearly with increasing catchment area with all parts of each field likely contributing sediment to the monitored edge-of-field outflows. When sediment flux was normalised to catchment area, ploughing during reseeding operations was the dominant factor which caused large increases in sediment yields. There were, however, contrasts in the erosion responses of the ploughed and reseeded fields with three catchments demonstrating a significant increase in sediment yield and one catchment exhibiting a negligible increase. Due to the identified importance of saturation-excess overland flow for the transport of detached particles, soil moisture was identified as a major factor controlling the erosion response of the studied field-scale catchments. Soil moisture is a key consideration for the viability and scheduling of ploughing and field operations (Robson and Thomasson, 1977; Schulte et al., 2012). Here, however, recent publications have highlighted that there is a paucity of information on the thresholds of soil moisture contents for safe tillage operations on different soils (Obour et al., 2017).

In response to this evidence gap, this study aimed to investigate the erosion response of the same set of fifteen grassland field scale catchments during scheduled ploughing and reseeding operations between 2012 and 2019. Such mechanistic understanding has important implications for supporting tailored risk management plans for reducing soil loss and protecting farm productivity. More specifically, the primary objectives were: (1) to examine the effects of scheduled ploughing and reseeding of lowland pasture on field scale sediment loss and; (2) to use the data assembled by this study to identify critical thresholds for soil moisture content for informing decisions pertaining to ploughing and reseeding of lowland pasture.

## 2 Materials and methods

### 2.1 Study site

**Fig. 1:**
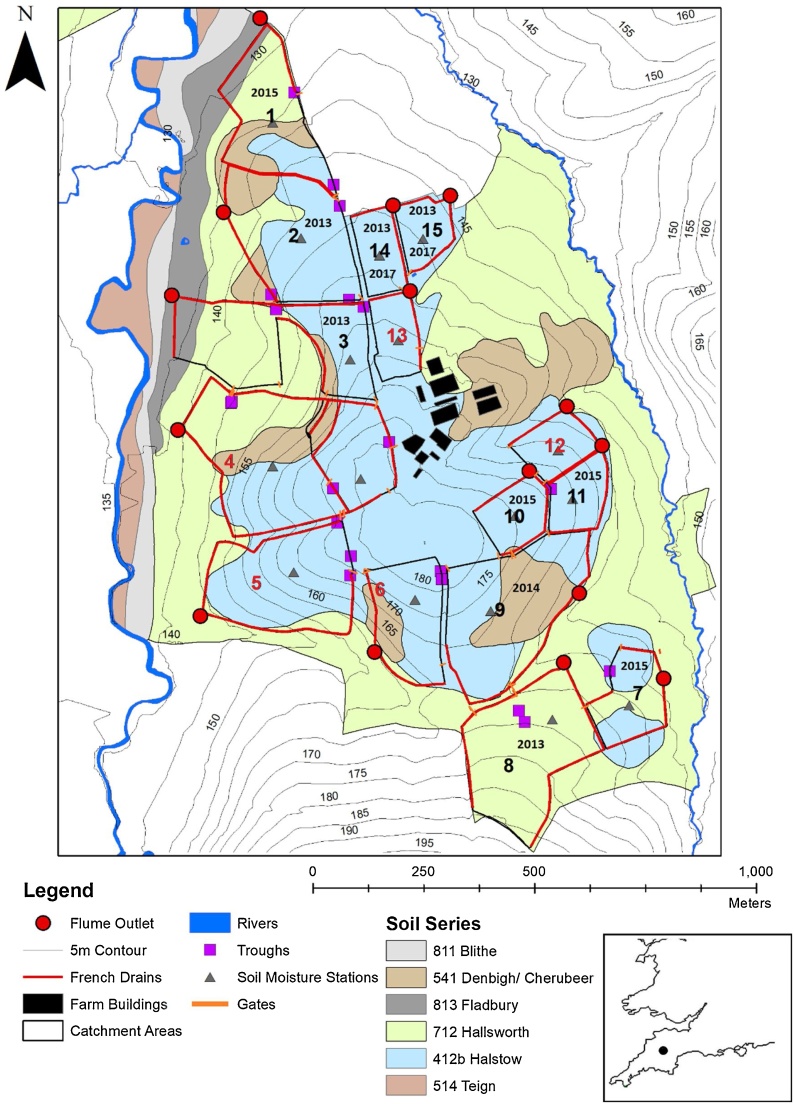
The North Wyke Farm Platform study area with catchment numbers; catchment numbers marked in red were not ploughed and reseeded and are therefore not included in this study; dates (i.e. year) of ploughing are shown within the boundaries of the individual catchments, modified from Pulley and Collins (2019).

The North Wyke Farm Platform (NWFP) is located in South West England in a lowland temperate landscape dominated by ruminant grazing. The platform is located on raised ground between the River Taw and one of its tributaries and consists of 15 field scale catchments (1.54–7.75 ha). Each catchment is hydrologically isolated by a network of 800 mm deep French drains backfilled with 20–50 mm clean granite and carbonate free, stone chips (Fig. 1; Table 1). The catchments have mean slopes ranging between 4.2 and 9.7 degrees, which due to the hilltop location of the site, have aspects varying in all directions. Soils exhibit slowly permeable drainage due to stony clay loam topsoils, meaning that the catchments are prone to seasonal waterlogging. Subsoils are poorly permeable with a greater clay content (60 % compared to 36 % for topsoils) and are composed of mottled stony clay. The presence of these soil profile characteristics supports the hydrological isolation.

**Table 1:** Characteristics of the 10 NWFP flume catchments which were ploughed and reseeded between 2013 and 2019.

Flume catchment	Name	Area (ha)	Mean slope (degrees)	Percentage of soil area^1^				Date ploughed	
				421b	712	541	813		Date reseeded
1	Pecketsford	4.81	5.83	22.8	41.4	25.5	10.3	28/07/2015	11/08/2015
2	Great Field	6.65	6.08	68	14.1	17.9	0	06/07/2013	30/07/2013
3	Poor Field	6.62	7.29	34.1	49.3	6	10.6	25/07/2014	21/08/2014
7	Lower Wyke Moor	2.6	7.54	57.5	42.5	0	0	22/07/2015	7/08/2015
8	Higher and Middle Wyke Moor	7.02	6.77	0.6	99.4	0	0	06/07/2013	31/07/2013 + 2/08/2013
9	Dairy Corner	7.75	8.42	56.9	5.8	37.3	0	27/07/2014	22/08/2014
10	Lower Wheaty	1.82	7.24	98.1	0	1.9	0	03/08/2015	11/08/2015
11	Dairy East	1.76	9.71	99.3	0.7	0	0	03/08/2015	12/08/2015
14	Longlands North	1.72	4.17	100	0	0	0	10/07/2013 (Phase 1) 07/09/2017 (Phase4)	7/08/2013 (phase 1) + 26/09/2017 (phase 4)
15	Longlands East	1.54	5.32	100	0	0	0	10/07/2013 (phase1) 02/10/2017 (phase 4)	7/08/2013 (phase 1) + 4/10/2017 (phase 4)

^1^soil definitions:

712, Hallsworth, Slowly permeable clayey soils often over shale. Some well drained fine loamy soils.

421b, Halstow, Slowly permeable clayey soils often over shale. Some well drained fine loamy soils.

541, Denbigh. Free draining permeable soils on hard (slate and shale) substrates with relatively low permeability and low storage capacity and Crediton Free draining permeable soils on soft sandstone substrates with relatively high permeability and high storage capacity.

813, Fladbury, Stoneless clayey soils, in places calcareous variably affected by groundwater. Flat land Risk of flooding.

The farm platform was set up to test the efficacy and sustainability of beef and sheep grazing systems (Takahashi et al., 2018). As such, scheduled ploughing and reseeding operations were conducted once or twice in 10 of the 15 catchments with the latter being split (5 catchments in each) between three farmlets testing sustainability trade-offs under: (1) business as usual long-term permanent pasture; (2) scheduled ploughing and reseeding for a high sugar grass monoculture, and (3) ploughing and reseeding for a grass/clover mix. As the primary purpose of the NWFP is not to study processes of soil erosion alone, scheduled ploughing and reseeding operations took place at different dates between 2013–2017 (Table 1). Phases 1–3 spanned 2013–2015, whereas phase 4 occurred in 2017. Catchments 2, 8, 14 and 15 were all ploughed and reseeded in 2013, catchments 3 and 9 in 2014, and catchments 1, 7, 10 and 11 in 2015. Catchments 14 and 15 were ploughed and reseeded again in 2017, giving the opportunity to compare flumes with different geographical characteristics ploughed at the same time (phases 1–3 spanning 2013–2015) as well as the same flumes ploughed at different times (i.e. 2013 – phase 1, 2014 - phase 2, 2015 – phase 3 and 2017 – phase 4) and, critically, during years with differing rainfall totals and soil moisture conditions. Each scheduled plough and reseed campaign generally comprised a sequence of conventional ploughing to a depth of 30 cm, power harrowing, Cambridge/ring rolling, fertiliser and lime application, power harrowing, flat rolling and seed drilling. Full details of the study site are provided in Orr et al. (2016) and Pulley and Collins (2019).

### 2.2 Methods

#### 2.2.1 Flume monitoring for sediment loss

Monitoring runoff and sediment emissions to water on the NWFP was facilitated by the network of French drains which converge on pre-collection chambers where samples of the runoff were collected. From the collection chambers, runoff moves to an open channel where discharge was recorded at 15-minute intervals using H-flumes (designed for a 1 in 50-year runoff event) installed originally with Tracom stage height gauges and Teledyne ISCO bubbler flow-meter devices (ISCO Open channel flow measurement handbook, 2001), but latterly with OTT hydromet pressure transducers. Turbidity was recorded in flow cells at 15-minute intervals. Up to 2016, turbidity was measured using YSI 6600V2 multiparameter sondes, but these were upgraded to EXO 2 sondes during that year. The NWFP has two complete sets of sensors for the EXO2 sondes, allowing one set to be calibrated in the laboratory whilst the other set is deployed in situ. The sets were rotated once every month, minimising downtime. Flow cells were installed because the multi-parameter sondes are vulnerable to drying as a consequence of intermittent field runoff. All sondes were calibrated quarterly on the basis of a two-point calibration; 0 (RO water) and 124 formazine nephelometric turbidity units (FNU). Automatic water samplers (ISCO) were deployed for the regular collection of water samples from the collection chambers, which after gravimetric filtration in the laboratory, were used to establish suspended sediment concentration-turbidity ratings for the turbidity sensors.

Turbidity time series were converted into suspended sediment concentration (SSC) with relationships developed using 100 mL samples of runoff from the flumes sampled over a range of flow conditions. All samples were filtered through 1.2 mg pore size glass fibre paper and oven dried at 105 °C for 60 min (Eq. 1; Bilotta et al., 2008; Peukert et al., 2014). For the autumn and winter months (i.e. up to 31/3 in the following year) post each phase of plough and reseed, an alternative rating (Eq. 2) was applied. Beyond the 31/3 of the year immediately following each phase of plough and reseed, Eq. 1 was applied again for the conversion of turbidity into SSC.

(1) SSC = 1.1804 * NTU + 0.0472 (r^2^ = 0.75)

(2) SSC = 0.7664 * NTU + 5.7116 (r^2^ = 0.91)

Since turbidity during flows of less than 0.2 L s^−1^ was not measured routinely due to inadequate water depth, the intercept value of the SSC-turbidity relationships was to infill these phases in the field discharge records. An ADCON RG1 tipping bucket rain gauge with 0.2 mm resolution and an Adcon SM1 soil moisture station have been installed in the centre of each catchment on the NWFP. Both rainfall and volumetric soil moisture are recorded at 15-minute intervals.

#### 2.2.2 Data analysis

Data analysis comprised two components. The first determined the impacts of scheduled ploughing and reseeding on suspended sediment concentrations, fluxes and yields. Bi-plots of SSC and flow before and after scheduled ploughing and reseeding were first produced for each of the 10 catchments to identify differences between the SSCs in field runoff at different flow rates. The sediment fluxes and yields for the ploughed and unploughed periods were then compared in the context of the duration and total rainfall of the post-plough periods. The proportion of the total 2013–2019 sediment flux which took place during the post-plough and reseed phases was then calculated for each flume to determine the impact of the plough and reseed phases on sediment yields measured during the entire monitoring period.

The second component of the data analysis determined the impact of soil moisture on post-plough and reseed sediment yields. The soil moisture time series was first examined to determine during which months of each year the soil was saturated and how this related to rainfall patterns. Histograms were then produced of the percentage of time, rainfall and sediment flux coincided with soil moisture contents (in 1% increments) to identify critical thresholds at which significant erosion occured. It was then identified how many days had passed from when the catchments were ploughed until the soil moisture threshold was exceeded. This was compared to the post-plough sediment yields to identify associated impacts. Finally, the time series covering the second post-plough phases (2017) for flumes 14 and 15, when exceptionally high erosion occurred, were examined to determine how sediment fluxes changed over the course of a post-plough period following an autumn rather than summer reseed campaign.

## 3 Results

### 3.1 The effects of scheduled ploughing and reseeding on sediment loss

**Fig. 2:**
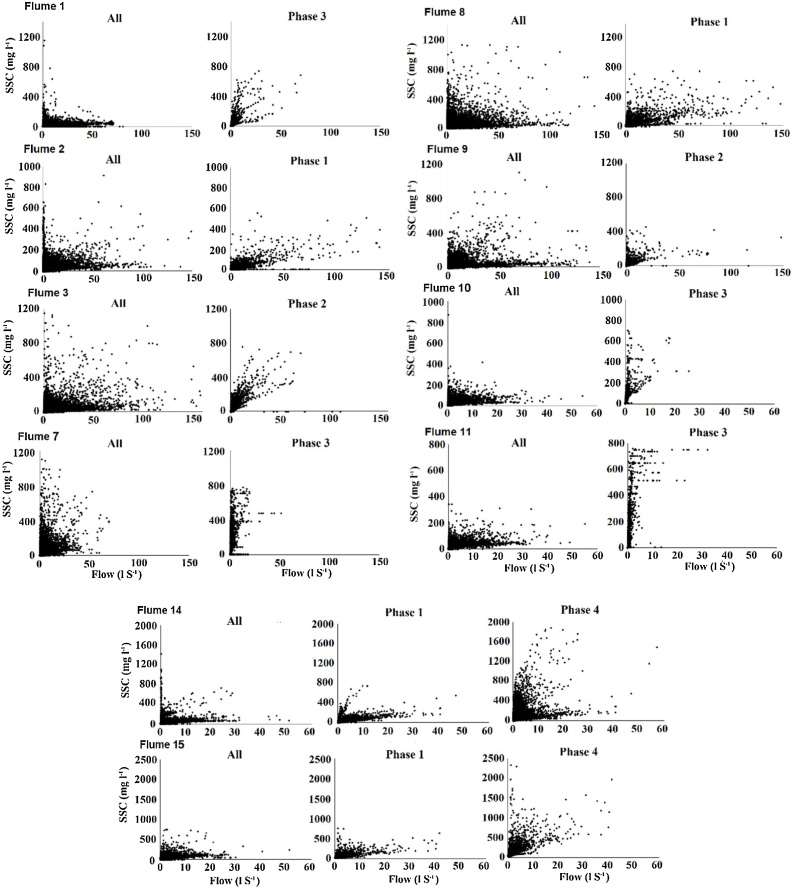
Bi-plots of flow and SSC in the runoff from each of the field scale catchments during the pre-plough and post-plough phases.

All flumes experienced an increase in SSC for a given flow rate after ploughing and over the autumn and winter to the following spring (31st March); however, the magnitude of the increase was highly variable between the flumes (Fig. 2). For Flumes 2, 7, 14 and 15 (ploughed 2013), the increases were small with SSCs mostly falling within the range found before ploughing and reseeding. At Flumes 10 and 11 (2015), and 14 and 15 (2017), SSCs were far higher than those during the un-ploughed phases. Flow and SSC were only weakly correlated due to a strong clockwise hysteresis in almost all flow events and high inter-event variability.

Precipitation and sediment yields were highly variable between the different flumes and post-plough phases (Table 2). Post-plough rainfall ranged from 461 to 1121 mm and sediment yields from 0.14 to 3.13 t. ha^−1^ yr^−1^. The post-plough and reseeding phases accounted for a very high proportion of total sediment fluxes despite only covering an average of 10.9 % of the 2002 days of flume monitoring. During the post-plough phases, sediment yields were also up to 5.5 times higher than the sediment yield for the entire duration of monitoring. For example, Flumes 14 and 15 experienced 78 % and 72 % of their total sediment fluxes during their two post-plough phases (Table 2). Plough and reseed phase 4 (2017) for Flumes 14 and 15 exhibited very high sediment yields (2.57 t. ha^−1^ yr^−1^ and 3.13 t. ha^−1^ yr^−1^) when compared to the responses at the other flumes (0.14 t. ha^−1^ yr^−1^ to 0.76 t. ha^−1^ yr^−1^) or indeed each other (0.72 t. ha^−1^ yr^−1^ and 0.73 t. ha^−1^ yr^−1^) during the first phase of pasture improvement (2013; Table 1). The flumes ploughed and reseeded in 2013 (2, 8, 14, 15) (phase1) had higher rainfall than the other ploughing and reseeding phases (Table 1), and with the exception of Flume 2, higher sediment yields. The highest sediment yields, however, were recorded during post-plough and reseed phase 4 (2017) at Flumes 14 and 15 which did not receive high rainfall during this particular post-plough period (Table 2).

**Table 2:** Rainfall, flow and sediment yields post-ploughing and reseeding.

Flume catchment	Post-plough rainfall (mm)	Post-plough sediment yield (t. ha^−1^ yr^-1^)	Total sediment yield (t. ha^−1^ yr^-1^)	Percentage of total sediment flux during the post-plough phases	Percentage of study duration in post plough phases	Post-plough / total sediment yield
1	461	0.48	0.12	31.29	7.89	3.98
2	1034	0.70	0.21	32.15	13.44	3.36
3	628	0.43	0.29	18.48	12.49	1.48
7	549	0.57	0.36	20.04	8.19	1.58
8	1121	0.89	0.35	34.74	13.44	2.59
9	651	0.14	0.15	11.55	12.39	0.93
10	473	0.30	0.10	22.07	7.59	2.91
11	483	0.76	0.15	38.51	7.59	5.09
14 (plough phase 1)	1067	0.72	0.50	19.21	13.24	1.45
15 (plough phase 1)	1046	0.73	0.60	16.22	13.24	1.23
14 (plough phase 2)	611	2.57	0.47	56.59	10.69	5.51
15 (plough phase 2)	538	3.13	0.60	45.86	10.69	5.23

There were variations in the sediment yields of catchments ploughed at the same time. For example, Flume 8 experienced a notably higher yield than Flume 2, and Flume 11 had a higher sediment yield than Flume 9. These differences were, however, far lower than those between the post-plough and reseed periods 1 and 4 (2013 and 2017) for Flumes 14 and 15 suggesting that the time at which a catchment was ploughed is of far greater importance than differences between field soil types, slope or morphology (Table 1).

### 3.2 Soil moisture as a critical control on sediment loss

**Fig. 3:**
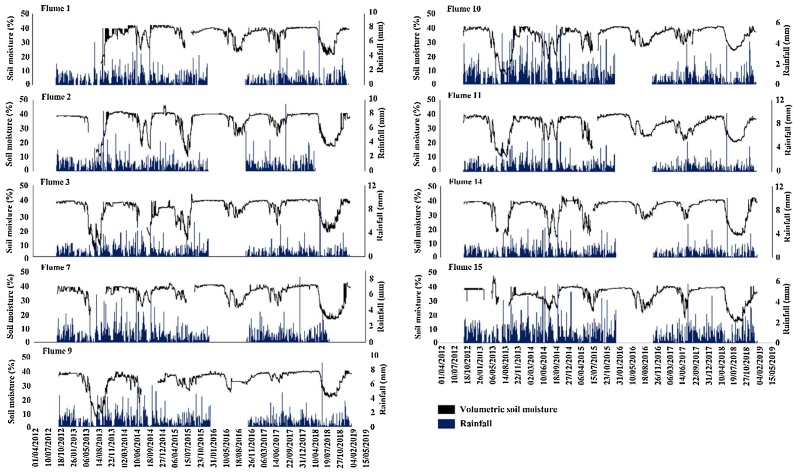
Soil moisture content and rainfall time series for the ploughed and reseeded catchments over the entire (2012 – 2019) study period.

During most summers soil moisture content was low; however, it increased quickly once significant rainfall resumed (Fig. 3). A single large storm event (>5 mm) was sometimes enough to cause a significant increase in soil moisture, such as those in late June and early October 2014. It was therefore the case that after many summer periods soil moisture was either already high or recovered to saturation within a short timescale. During other dry periods, soil moisture was slower to recover, such as after the summer of 2013 where the soil did not become saturated until late November. The most prolonged dry period was in the summer of 2018 where low rainfall resulted in a soil moisture deficit extending into February 2019. The summers of both 2012 and 2017 were notable as high rainfall resulted in soil moisture content remaining close to saturation throughout the entire year.

**Fig. 4:**
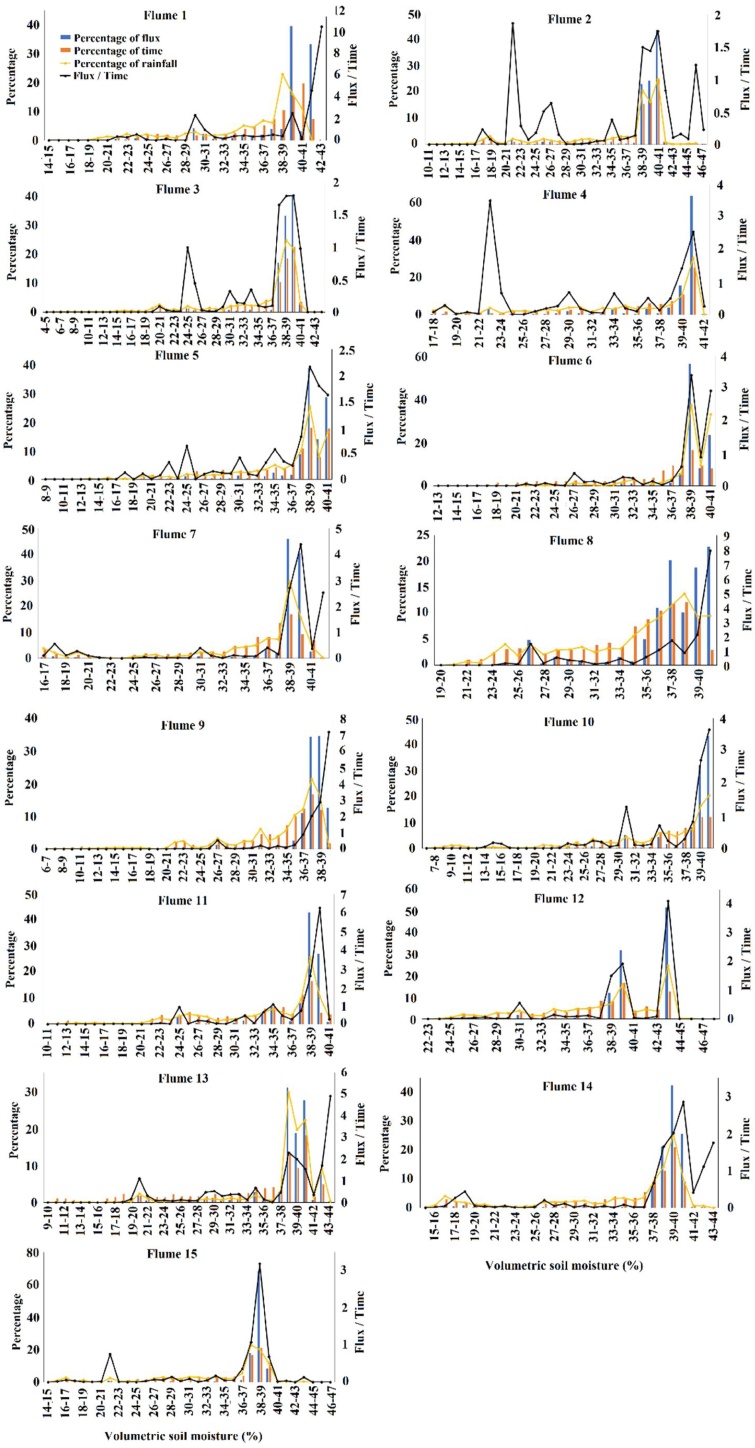
Histograms of the percentage of total rainfall, sediment flux, and proportion of time at different soil moisture thresholds for all 10 ploughed and reseeded catchments.

Due to the rapid recovery of soil moisture after the dry summers, the majority of the study period was spent with soil moisture content close to saturation in all catchments (Fig. 4). Most of the rainfall and sediment flux therefore took place during such field conditions. A distinct soil moisture threshold over which a significant proportion of the total sediment flux occurred could be identified for each catchment (Fig. 4). This threshold was at 37–38 % soil volumetric moisture content for all flumes, apart from Flume 8, where it was lower at 35–36 %. There was a higher sediment flux per unit time and rainfall with greater soil moisture content, even after the soil moisture-based erosion threshold was passed. When soil moisture was below these thresholds, sediment flux was very low, likely due to the absence of raindrop-impacted saturation-excess overland flow to transport detached soil particles.

**Fig. 5:**
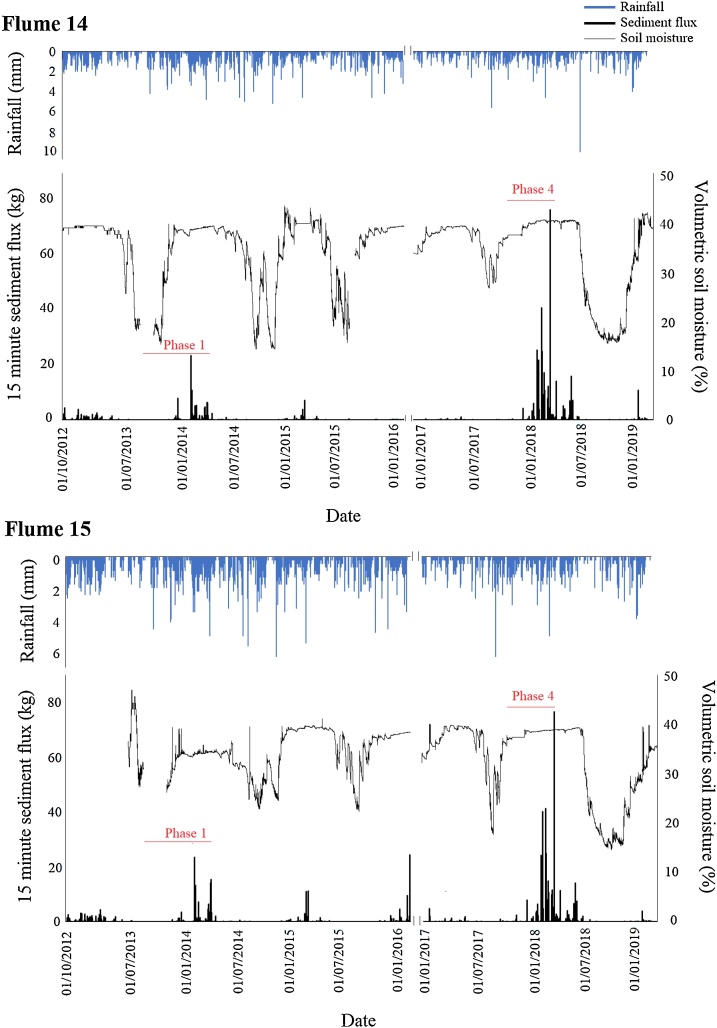
Rainfall, volumetric soil moisture content and 15-minute sediment flux time series for Flumes 14 and 15 over the entire (2012 – 2019) study period. Note data was lost during 2016.

The differences between sediment fluxes in the non-ploughed and two post plough (2013 and 2017) phases for Flume 14 can be clearly seen in Fig. 5. Even during the post-plough and reseed phase 1, when soil moisture thresholds were reached 85 days after ploughing, sediment fluxes were higher than during the non-plough period. When the soils were ploughed with soil moisture above the erosion threshold in plough and reseed phase 4 sediment fluxes were far higher than during the first plough and reseed period. It can clearly be observed that intense rainfall during the first half of the post-plough period of phase 1 resulted in negligible sediment flux due to low soil moisture content (Fig. 5).

**Fig. 6:**
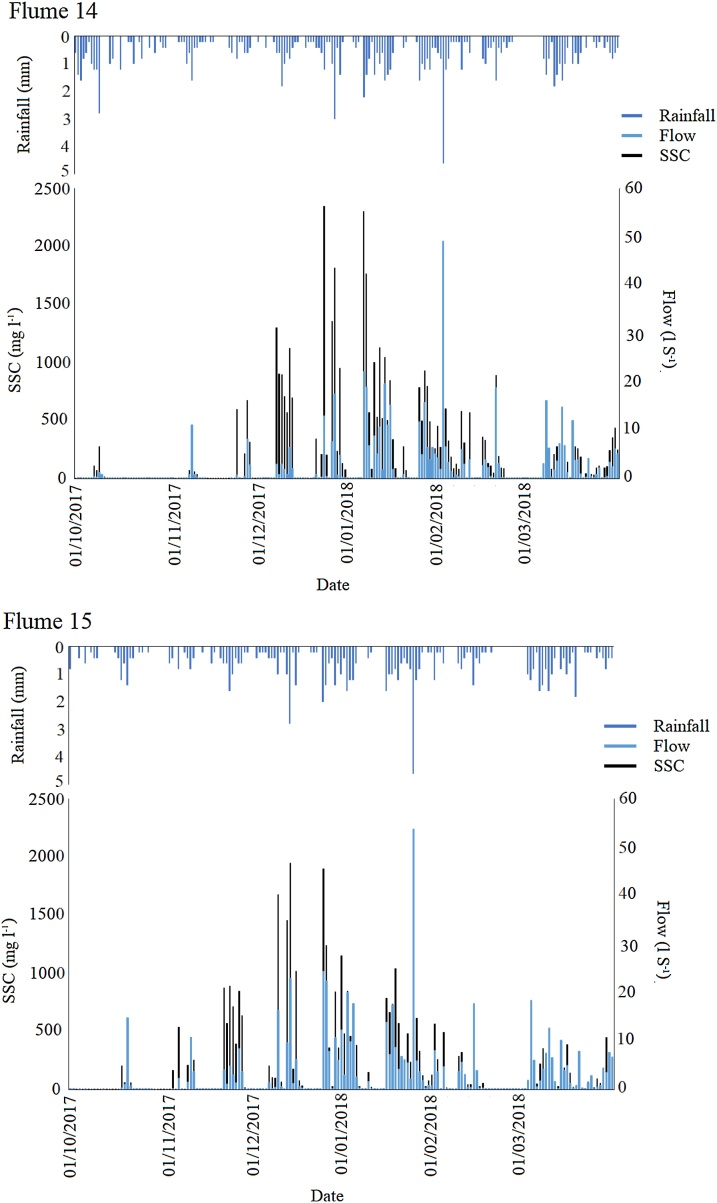
Flume 14 and 15 time series focussing on the 2017-2019 time period post-plough and reseed phase 4.

The large increase in sediment yield for Flumes 14 and 15 following plough and reseed phase 4 (2017) was not repeated in the other flumes even when as little as 20 days had elapsed between rainfall and soil moisture reaching the erosion threshold (Table 3). This may be a result of the timing of ploughing operations as during summer months grassland cover may establish quickly. In contrast October ploughing in Flumes 14 and 15 with lower temperatures and light intensity will likely cause grass cover to take longer to establish. Flumes 11 and 10 were ploughed in the summer and had only 42 and 20 days until soil moisture thresholds were reached but did not exhibit an extreme erosion response like the autumn ploughed flumes. Over the course of the time period (autumn 2017 – spring 2018) spanning post-plough and reseed phase 4 in Flumes 14 and 15, the SSC for a given rainfall or flow rate decreased over time (Fig. 6). This may similarly represent the effects of sward re-establishment or the depletion of easily detachable particles resulting in a less erodible soil surface.

**Table 3:** Soil moisture characteristics of the ploughed and reseeded catchments.

Flume catchment	Plough date	Soil moisture erosion threshold (%)	Soil moisture on plough date (volumetric %)	Number of days from ploughing to reaching erosion moisture threshold	Total duration of post-plough phase
1	28/07/2015	39	26.6	124	158*
2	06/07/2013	38	14.0	111	269
3	25/07/2014	37	29.9	111	250
7	22/07/2015	38	28.1	54	164*
8	06/07/2013	35	25.3	114	269
9	27/07/2014	36	24.3	172	248
10	03/08/2015	38	24.0	42	152*
11	03/08/2015	37	16.4	20	152*
14 (phase 1)	10/07/2013	37	19.3	85	265
15 (phase 1)	10/07/2013	37	31.0	85	265
14 (phase 4)	07/09/2017	37	37.0	0	214
15 (phase 4)	02/10/2017	37	37.2	0	214

* Equipment failure in January 2016 resulted in the partial loss of data.

## 4 Discussion

Recent publications have highlighted that there is a paucity of information on the thresholds of soil moisture contents for safe tillage operations on different soils (Obour et al., 2017). The results generated by our study here, provide such thresholds of 35–38 % for the soils on the NWFP which are typical of many soil types with slowly permeable drainage in temperate climates. The results presented herein underscore the significance of soil moisture content in substantially elevating the risk of soil erosion and sediment loss from long-term pasture fields during ploughing and reseeding for sward improvement. Annual cycles of soil moisture content therefore represent a key consideration for both the viability and scheduling of such farm management practices (Robson and Thomasson, 1977; Schulte et al., 2012).

Using available monitoring data, a typical suspended sediment yield for UK catchments has been estimated at 0.5 t ha^−1^ yr^−1^ (Walling and Webb, 1987). A suspended sediment yield for the upper River Exe, which is approximately 20 km to the east of the study site, has been calculated at 0.19 t. ha^−1^ yr^−1^ and a yield of 0.26 t. ha^−1^ yr^−1^ was calculated for the nearby River Clyst (Walling and Webb, 1987). A much higher yield of 0.89 t. ha^−1^ yr^−1^ was calculated for the River Torridge which is approximately 10 km to the west of the study catchment Nicholls (2000). The overall mean sediment yield for all fields during the entire (2012–2019) monitoring period was calculated at 0.33 t. ha^−1^ yr^−1^. However, after autumn ploughing, the measured post-plough yields of 2.57 t. ha^−1^ yr^−1^ and 3.13 t. ha^−1^ yr^−1^ illustrate how ploughed pasture fields can represent a significant source of sediment inputs to rivers. It is important to consider that these elevated yields only represent the higher risk wet winter months and exclude drier summer months. The mean post-plough sediment yield of 0.58 t. ha^−1^ yr^−1^ for the catchments ploughed in summer months represents an elevation over that for the entire (2012–2019) monitoring period on the farm platform, but remains more comparable to UK typical suspended sediment yields at catchment scale. When considering connectivity between fields and river channels, it is unlikely that all eroded sediment exported from high risk fields will reach the river channel, but if ploughing and reseeding of grassland is undertaken in autumn when soil moisture contents can be higher, the mass of sediment reaching rivers will be higher.

Farm advisors typically use soil wetness class and land capability classes to deliver guidance on land management with the aim of reducing losses of sediment and nutrients to water courses. Soil wetness classes are used to reflect the average annual duration of soil profile waterlogging (Lilly and Matthews, 1994). Land capability is founded on the interaction between soil water regime and land use flexibility, as captured by the national Soil Survey (Bibby et al., 1982). Soils on the NWFP are land capability classes 4 in the main, but also 5, meaning that there are severe soil wetness driven limitations for farming operations and crop types. In many cases, farmers make intuitive assessments of soil workability for ploughing and seedbed preparation, but such approaches are very subjective and run the risk of failure (Cadena-Zapata, 1999; Cadena-Zapata et al., 2002; Obour et al., 2017). Soils are classed as workable for ploughing and tillage operations when cultivation and seedbed preparation avoid smearing and compaction (Rounsevell and Jones, 1993; Müller et al., 2011). Self-assessment in the field typically involves a manual plastic limit test in which soil is pressed, remoulded and rolled by the hand to assess its consistency as a proxy for soil moisture content (Müeller et al., 2003). Such self-assessment can be unpopular as it is time-consuming (Edwards et al., 2016; Obour et al., 2017). Scientific studies such as the one reported here can at least provide some guideline data for specific soils and hydroclimatic settings, but such data need to be coupled with on-farm manual assessments. Full ploughing and reseeding provides an opportunity to maximise benefit from advances in grass plant breeding (Reheul et al., 2017). Such operations are undertaken in spring or summer/early autumn to ensure sufficient soil moisture content and light for germination (Frame, 1992; Haugland and Froud-Williams, 2001; Kayser et al., 2018). Renovation during spring has been shown to be less impactful on alternative externalities to water such as nitrogen emissions, especially with well-scheduled tillage and well-planned fertilizer applications, since grass growth utilise mineralised nitrogen prior to the onset of the winter drainage phase (Silgram and Shepherd, 1999; Shepherd et al., 2001; Seidel et al., 2007, 2009; Velthof et al., 2010). Some experimental evidence suggests that with well-planned fertilization, the lifespan of a productive sward can be increased, reducing the frequency of ploughing and reseeding (Loiseau et al., 1992). For soil erosion and sediment loss, and indeed additional externalities not covered in the work reported here, ploughing and reseeding during unsuitably high soil moisture levels is clearly the most destructive form of grassland renovation (Skinner and Chambers, 1996; Velthof et al., 2010; MacDonald et al., 2011) and so decisions to proceed must be taken carefully in the context of prevailing weather and soil moisture conditions. Such severe renovation will not deliver production and environmental benefits if management issues such as overstocking, inappropriate grazing regimes and soil management are not addressed post ploughing and reseeding.

Whilst our work identifies that the proper timing of plough and reseeding operations can reduce soil loss, the increased sediment yields which occurred even after summer ploughing, highlights the importance of alternatives to ploughing and reseeding. A long-term permanent sward is typically less productive than a physically reseeded sward (Dubeux et al., 2007; Radrizzani et al., 2010; Creighton et al., 2011). Grassland renovation is typically a reaction to declining sward yield and changes in botanical composition including entry of weeds as well as reduced vegetation cover (Frame, 1992; Taube and Conijn, 2004; Kayser et al., 2018). Where soil compaction is a primary cause of reduced sward production, subsoiling can be used to help aerate and regenerate soil, thereby avoiding the need for complete ploughing and reseeding (Hopkins and Wilkins, 2006). Alternatively, strip-seeding can also be used to target regeneration of the sward (BAL, 1991; Williams and Hayes, 1990). Despite such alternatives, ploughing and reseeding remain commonplace, meaning that the risk of elevated soil erosion and sediment loss is high during sub-optimal field conditions. The critical role of soil moisture content in driving unintended consequences of field operations in lowland pasture systems highlighted by this work is therefore of importance to guiding the timing of field operations.

## 5 Conclusions

Farm productivity and the avoidance of long-term environmental damage both on-site and off-site is dependent upon tailored risk management. This study illustrates the importance of prevailing soil moisture content in the context of managing soil erosion risk accompanying scheduled pasture ploughing and reseeding. Ploughing was shown to result in a higher sediment yield during the following autumn and winter months when compared to winters when fields were left unploughed. Overall, a high proportion (mean 28.8 %) of total monitored (2012–2019) sediment fluxes occurred during these post-plough periods despite them only covering an average of 10.9 % of the 2002 days of flume monitoring. The largest increases in erosion took place when soils were ploughed late in October when soils were saturated, with subsequent autumn and winter sediment yields calculated at 2.57 t. ha^−1^ yr^−1^ and 3.13 t. ha^−1^ yr^−1^. Yields for the same catchments after summer ploughing were 0.72 t. ha^−1^ yr^−1^ and 0.73 t. ha^−1^ yr^−1^. Thresholds of 35–38 % soil moisture were identified at which ploughing represented a highly elevated erosion risk. There was, however, evidence of a reduction in sediment yields over the course of the post-plough periods likely due to the re-establishment of sward cover. As a result, after summer ploughing as little as 20 days between operations and soil moisture reaching the erosion threshold was enough time to prevent extreme erosion rates. It is likely that colder temperatures and lower light levels slow sward reestablishment after autumn ploughing contributing to prolonged exposure of topsoils to erosion and the concomitant monitored high sediment yields.

Elevated soil moisture content reduces the readiness of soils for tillage and thereby their capacity to withstand field operations without soil structural damage and both economic and environmental consequence. Our results confirm that soil moisture is the single most important factor controlling erosion during rainfall events, since it affects soil structure and hydraulic response. Although intermittent ploughing and reseeding represents the single most significant management shock in intensively managed grazing systems, sward growth means that even this extreme form of management is less destructive over time than conversion to arable cropping with annual schedules of tillage and sowing.

## Declaration of Competing Interest

The authors declare that they have no known competing financial interests or personal relationships that could have appeared to influence the work reported in this paper.
